# Identification of *Corynebacterium jeikeium* from a CAPD patient by *16S* rDNA PCR – a case report

**DOI:** 10.1186/1471-2334-14-S3-P34

**Published:** 2014-05-27

**Authors:** Gopalakrishnan Sathya Narayanan, Radha Vijayaraghavan, Thangam Menon

**Affiliations:** 1 Department of Microbiology, Dr.AL Mudaliar Post Graduate Institute of Basic Medical Sciences, University of Madras, Taramani, Chennai, India; 2 Department of Nephrology, Southern Railway Hospital, Perambur - ICF, Chennai, India

## Background

Peritonitis is a serious complication in patients who are on continuous ambulatory peritoneal dialysis (CAPD). We report a rare case of peritonitis caused by *Corynebacteriumjeikeium*.

## Case report

A 51-year-old male was admitted in Southern Railway Hospital, Chennai with complaint of abdominal pain for four days. He had a history of renal failure two years before admission and was started on CAPD for end stage renal disease. Prior to admission he was on ambulatory peritoneal dialysis (APD). Peritoneal dialysis (PD) fluid was collected and transported to laboratory aseptically. Peritoneal fluid was cloudy at the time of collection. It was inoculated into BHIB culture bottles which were incubated at 37° C and inspected every day for turbidity. DNA was extracted and analyzed for presence of *16S* rDNA by PCR. The bacterial culture and identification was done as per standard procedures. Antibiotic susceptibility test was performed for the isolate. DNA was extracted from the colonies and analyzed for the presence of *16S* rDNA by PCR and amplicons were sequenced (Xcelris Laboratories, Ahmedabad).The *16S* rDNA FASTA sequence was subjected to BLAST analysis and it was confirmed as *Corynebacterium jeikeium*. (Genbank Accession No. KF909134). The isolate was found to be multi drug resistant and sensitive only to vancomycin. The patient responded well to therapy.

## Conclusion

Precise and rapid identification of these potentially multi drug resistant organisms are important for patient management. *16S* rDNA PCR was found to be a useful tool in establishing etiology in cases of infections caused by rare pathogens.

